# 5-(Adamantan-1-yl)-3-[(2-meth­oxy­eth­yl)sulfan­yl]-4-phenyl-4*H*-1,2,4-triazole

**DOI:** 10.1107/S1600536812029510

**Published:** 2012-07-04

**Authors:** Ali A. El-Emam, Ebtehal S. Al-Abdullah, Hanadi H. Asiri, Suchada Chantrapromma, Hoong-Kun Fun

**Affiliations:** a Department of Pharmaceutical Chemistry, College of Pharmacy, King Saud University, PO Box 2457, Riyadh 11451, Saudi Arabia; b Crystal Materials Research Unit, Department of Chemistry, Faculty of Science, Prince of Songkla University, Hat-Yai, Songkhla 90112, Thailand; c X-ray Crystallography Unit, School of Physics, Universiti Sains Malaysia, 11800 USM, Penang, Malaysia

## Abstract

In the title adamantyl derivative, C_21_H_27_N_3_OS, the terminal meth­oxy­ethyl unit is disordered over two orientations with a refined site-occupancy ratio of 0.846 (6):0.154 (6). The 1,2,4-triazole ring is statistically planar [r.m.s. deviation = 0.002 (2) Å] and the phenyl substituent is almost perpendicular to its mean plane [dihedral angle = 83.57 (11)°]. No directional inter­molecular inter­actions were observed in the crystal structure.

## Related literature

For the biological activity of adamantane derivatives, see: Kadi *et al.* (2010 ▶). For related adamantyl-1,2,4-triazole structures, see: Al-Abdullah *et al.* (2012 ▶); Almutairi *et al.* (2012 ▶); El-Emam *et al.* (2012 ▶). For substituted sulfanyl-1,2,4-triazole structures, see: Fun *et al.* (2011 ▶); Wang *et al.* (2011 ▶).
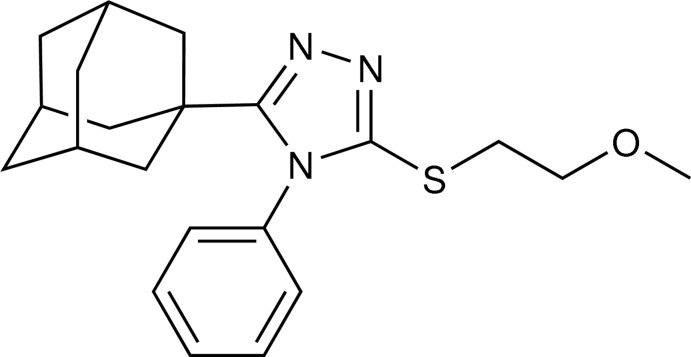


## Experimental

### 

#### Crystal data

C_21_H_27_N_3_OS
*M* *_r_* = 369.53
Monoclinic, 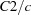

*a* = 22.5107 (5) Å
*b* = 9.7642 (2) Å
*c* = 19.5594 (3) Å
β = 116.679 (1)°
*V* = 3841.43 (13) Å^3^
*Z* = 8
Cu *K*α radiation
μ = 1.60 mm^−1^
*T* = 296 K
0.59 × 0.56 × 0.18 mm

#### Data collection

Bruker SMART APEXII CCD diffractometer
Absorption correction: multi-scan (*SADABS*; Bruker, 2009 ▶) *T*_min_ = 0.453, *T*_max_ = 0.761
12922 measured reflections
3499 independent reflections
3075 reflections with *I* > 2σ(*I*)
*R*_int_ = 0.028

#### Refinement

*R*[*F* ^2^ > 2σ(*F* ^2^)] = 0.045
*wR*(*F* ^2^) = 0.130
*S* = 1.05
3499 reflections
245 parameters
H-atom parameters constrained
Δρ_max_ = 0.22 e Å^−3^
Δρ_min_ = −0.30 e Å^−3^

### 

Data collection: *APEX2* (Bruker, 2009 ▶); cell refinement: *SAINT* (Bruker, 2009 ▶); data reduction: *SAINT*; program(s) used to solve structure: *SHELXTL* (Sheldrick, 2008 ▶); program(s) used to refine structure: *SHELXTL*; molecular graphics: *SHELXTL*; software used to prepare material for publication: *SHELXTL* and *PLATON* (Spek, 2009 ▶).

## Supplementary Material

Crystal structure: contains datablock(s) global, I. DOI: 10.1107/S1600536812029510/hb6881sup1.cif

Structure factors: contains datablock(s) I. DOI: 10.1107/S1600536812029510/hb6881Isup2.hkl

Supplementary material file. DOI: 10.1107/S1600536812029510/hb6881Isup3.cml

Additional supplementary materials:  crystallographic information; 3D view; checkCIF report

## Figures and Tables

**Fig. 1.**
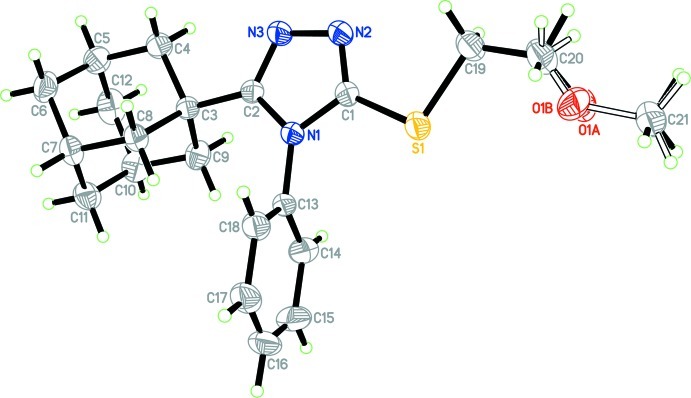
The molecular structure of the title compound, showing 30% probability displacement ellipsoids. Open bonds show the minor disorder component.

**Fig. 2.**
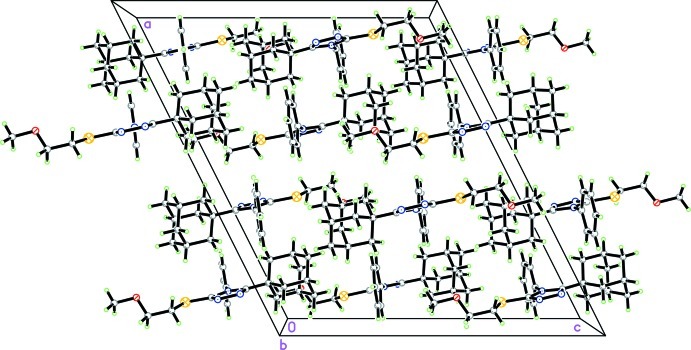
The crystal packing of the title compound viewed along the b axis. Only the major component was shown.

## supplementary crystallographic information

### Comment

In continuation of our interest in the chemical and pharmacological properties of adamantane derivatives (Kadi *et al.*, 2010), we synthesized the title compound (I) as a potential chemotherapeutic agent and herein its crystal structure is reported.

In the molecule of the title adamantyl derivative, C_21_H_27_N_3_OS, (Fig. 1) the terminal methoxyethyl unit is disordered over two orientations with the refined site-occupancy ratio of 0.845 (6):0.155 (6). The 1,2,4-triazole ring is planar with an *r.m.s*. deviation of 0.002 (2) Å. The phenyl substituent is almost perpendicular to the mean plane of the 1,2,4-triazole ring with the dihedral angle of 83.57 (11)°. The adamantyl group is planarly attached to the 1,2,4-triazole ring at atom position 5 or atom C2. The 2-(methoxyethyl)sulfanyl substituent is planarly attached to this five-membered ring with the torsion angle C1–S1–C19–C20 = 179.61 (15)°. The orientation of the terminal disordered methoxyethyl unit can be indicated by the torsion angles C21–O1–C20–C19 = -176.1 (2)° for the major component (A) and 127.7 (7)° for the minor component (B). The bond distances in (I) are comparable with those in related structures (Al-Abdullah *et al.*, 2012; Almutairi *et al.*, 2012; El-Emam *et al.*, 2012; Fun *et al.*, 2011 and Wang *et al.*, 2011).

Even though no intermolecular hydrogen bond was observed in the crystal packing of (I), however the crystal packing was shown in Fig. 2 to illustrate the arrangement of the molecules.

### Experimental

A mixture of 3-(adamantan-1-yl)-4-phenyl-4*H*-1,2,4-triazole-5-thiol (623 mg, 2 mmol), 1-bromo-2-methoxyethane (278 mg, 2 mmol) and anhydrous potassium carbonate (276 mg, 2 mmol) in *N*,*N*-dimethylformamide (5 ml) was stirred at room temperature for 24 h. Water (15 ml) was added and the mixture was stirred for 30 min. The separated crude product was filtered, washed with water, dried and crystallized from aqueous ethanol to yield 196 gm (53 %) of the title compound as colorless plate-shaped crystals, M.p. 428-430 K.

### Refinement

All H atoms were placed in calculated positions with d(C-H) = 0.93 Å for aromatic (phenyl), 0.98 Å for aromatic (adamantyl), 0.97 Å for CH_2_ and 0.96 Å for CH_3_ atoms. The *U*_iso_ values were constrained to be 1.5*U*_eq_ of the carrier atom for methyl H atoms and 1.2*U*_eq_ for the remaining H atoms. The terminal methoxyethyl unit is disordered over two sites with refined site occupancies of 0.846 (6) and 0.154 (6).

### Crystal data

**Table:**

C_21_H_27_N_3_OS	*F*(000) = 1584
*M**_r_* = 369.53	*D*_x_ = 1.278 Mg m^−^^3^
Monoclinic, *C*2/*c*	Melting point = 428–430 K
Hall symbol: -C 2yc	Cu *K*α radiation, λ = 1.54178 Å
*a* = 22.5107 (5) Å	Cell parameters from 3499 reflections
*b* = 9.7642 (2) Å	θ = 5.0–69.0°
*c* = 19.5594 (3) Å	µ = 1.60 mm^−^^1^
β = 116.679 (1)°	*T* = 296 K
*V* = 3841.43 (13) Å^3^	Plate, colorless
*Z* = 8	0.59 × 0.56 × 0.18 mm

### Data collection

**Table:**

Bruker SMART APEXII CCD diffractometer	3499 independent reflections
Radiation source: fine-focus sealed tube	3075 reflections with *I* > 2σ(*I*)
Graphite monochromator	*R*_int_ = 0.028
φ and ω scans	θ_max_ = 69.0°, θ_min_ = 5.0°
Absorption correction: multi-scan (*SADABS*; Bruker, 2009)	*h* = −27→26
*T*_min_ = 0.453, *T*_max_ = 0.761	*k* = −11→11
12922 measured reflections	*l* = −23→23

### Refinement

**Table:**

Refinement on *F*^2^	Primary atom site location: structure-invariant direct methods
Least-squares matrix: full	Secondary atom site location: difference Fourier map
*R*[*F*^2^ > 2σ(*F*^2^)] = 0.045	Hydrogen site location: inferred from neighbouring sites
*wR*(*F*^2^) = 0.130	H-atom parameters constrained
*S* = 1.05	*w* = 1/[σ^2^(*F*_o_^2^) + (0.0809*P*)^2^ + 1.3248*P*] where *P* = (*F*_o_^2^ + 2*F*_c_^2^)/3
3499 reflections	(Δ/σ)_max_ = 0.001
245 parameters	Δρ_max_ = 0.22 e Å^−^^3^
0 restraints	Δρ_min_ = −0.30 e Å^−^^3^

### Special details

**Table:**

Geometry. All esds (except the esd in the dihedral angle between two l.s. planes) are estimated using the full covariance matrix. The cell esds are taken into account individually in the estimation of esds in distances, angles and torsion angles; correlations between esds in cell parameters are only used when they are defined by crystal symmetry. An approximate (isotropic) treatment of cell esds is used for estimating esds involving l.s. planes.
Refinement. Refinement of F^2^ against ALL reflections. The weighted R-factor wR and goodness of fit S are based on F^2^, conventional R-factors R are based on F, with F set to zero for negative F^2^. The threshold expression of F^2^ > 2sigma(F^2^) is used only for calculating R-factors(gt) etc. and is not relevant to the choice of reflections for refinement. R-factors based on F^2^ are statistically about twice as large as those based on F, and R- factors based on ALL data will be even larger.

### Fractional atomic coordinates and isotropic or equivalent isotropic displacement parameters (Å^2^)

**Table:**

	*x*	*y*	*z*	*U*_iso_*/*U*_eq_	Occ. (<1)
S1	0.08715 (2)	0.28171 (4)	0.23891 (2)	0.05409 (17)	
N1	0.11945 (7)	0.33435 (13)	0.38845 (7)	0.0413 (3)	
N2	0.10327 (8)	0.11543 (14)	0.35822 (9)	0.0532 (4)	
N3	0.11921 (8)	0.13061 (14)	0.43546 (9)	0.0526 (4)	
O1A	0.10717 (11)	0.1555 (2)	0.10375 (10)	0.0672 (7)	0.846 (6)
O1B	0.0543 (8)	0.1948 (10)	0.0738 (6)	0.076 (5)	0.154 (6)
C1	0.10350 (8)	0.23789 (17)	0.33203 (9)	0.0444 (4)	
C2	0.12860 (8)	0.26011 (16)	0.45299 (9)	0.0424 (3)	
C3	0.14495 (8)	0.31590 (16)	0.53129 (9)	0.0417 (3)	
C4	0.13939 (12)	0.19832 (18)	0.58035 (11)	0.0593 (5)	
H4A	0.0946	0.1616	0.5565	0.071*	
H4B	0.1698	0.1254	0.5835	0.071*	
C5	0.15593 (12)	0.2492 (2)	0.66116 (11)	0.0655 (5)	
H5A	0.1524	0.1727	0.6916	0.079*	
C6	0.10653 (11)	0.3612 (2)	0.65583 (11)	0.0627 (5)	
H6A	0.0616	0.3249	0.6320	0.075*	
H6B	0.1161	0.3931	0.7067	0.075*	
C7	0.11237 (9)	0.47934 (19)	0.60849 (10)	0.0529 (4)	
H7A	0.0812	0.5520	0.6053	0.063*	
C8	0.09607 (8)	0.42908 (18)	0.52755 (9)	0.0463 (4)	
H8A	0.0990	0.5050	0.4972	0.056*	
H8B	0.0510	0.3938	0.5030	0.056*	
C9	0.21600 (9)	0.3739 (2)	0.57084 (11)	0.0568 (4)	
H9A	0.2475	0.3035	0.5738	0.068*	
H9B	0.2201	0.4496	0.5411	0.068*	
C10	0.23171 (9)	0.4233 (2)	0.65161 (11)	0.0649 (5)	
H10A	0.2770	0.4604	0.6762	0.078*	
C11	0.18282 (10)	0.5350 (2)	0.64632 (11)	0.0629 (5)	
H11A	0.1932	0.5681	0.6972	0.076*	
H11B	0.1866	0.6113	0.6167	0.076*	
C12	0.22647 (12)	0.3049 (3)	0.69881 (11)	0.0755 (6)	
H12A	0.2375	0.3359	0.7502	0.091*	
H12B	0.2575	0.2333	0.7022	0.091*	
C13	0.12558 (8)	0.47952 (16)	0.37871 (9)	0.0433 (4)	
C14	0.18701 (10)	0.5351 (2)	0.39749 (12)	0.0607 (5)	
H14A	0.2247	0.4800	0.4152	0.073*	
C15	0.19180 (14)	0.6760 (2)	0.38947 (15)	0.0795 (7)	
H15A	0.2331	0.7154	0.4022	0.095*	
C16	0.13615 (16)	0.7570 (2)	0.36300 (13)	0.0793 (7)	
H16A	0.1399	0.8511	0.3589	0.095*	
C17	0.07556 (13)	0.6996 (2)	0.34282 (12)	0.0694 (6)	
H17A	0.0379	0.7549	0.3241	0.083*	
C18	0.06922 (10)	0.55996 (19)	0.34984 (10)	0.0544 (4)	
H18A	0.0276	0.5210	0.3353	0.065*	
C19	0.07046 (11)	0.1108 (2)	0.19793 (11)	0.0597 (5)	
H19A	0.1092	0.0533	0.2248	0.072*	
H19B	0.0337	0.0708	0.2040	0.072*	
C20	0.05380 (13)	0.1161 (3)	0.11512 (13)	0.0738 (6)	
H20A	0.0389	0.0263	0.0926	0.089*	0.846 (6)
H20B	0.0175	0.1800	0.0894	0.089*	0.846 (6)
H20C	0.0797	0.0424	0.1098	0.089*	0.154 (6)
H20D	0.0087	0.0837	0.0911	0.089*	0.154 (6)
C21	0.08943 (16)	0.1691 (3)	0.02410 (14)	0.0851 (7)	
H21A	0.1278	0.1967	0.0183	0.128*	0.846 (6)
H21B	0.0552	0.2370	0.0017	0.128*	0.846 (6)
H21C	0.0735	0.0829	−0.0011	0.128*	0.846 (6)
H21D	0.0838	0.2477	−0.0077	0.128*	0.154 (6)
H21E	0.1359	0.1547	0.0565	0.128*	0.154 (6)
H21F	0.0711	0.0899	−0.0075	0.128*	0.154 (6)

### Atomic displacement parameters (Å^2^)

**Table:**

	*U*^11^	*U*^22^	*U*^33^	*U*^12^	*U*^13^	*U*^23^
S1	0.0784 (3)	0.0442 (3)	0.0448 (3)	0.00191 (19)	0.0322 (2)	−0.00132 (15)
N1	0.0540 (7)	0.0328 (6)	0.0422 (7)	0.0010 (5)	0.0260 (6)	0.0000 (5)
N2	0.0774 (10)	0.0377 (7)	0.0523 (8)	−0.0018 (6)	0.0360 (8)	−0.0030 (6)
N3	0.0776 (10)	0.0362 (7)	0.0533 (8)	0.0003 (6)	0.0376 (8)	0.0005 (6)
O1A	0.0721 (14)	0.0758 (13)	0.0534 (10)	−0.0075 (10)	0.0280 (10)	−0.0151 (8)
O1B	0.123 (13)	0.055 (5)	0.065 (6)	0.010 (6)	0.056 (7)	0.022 (5)
C1	0.0529 (8)	0.0404 (8)	0.0449 (8)	0.0008 (6)	0.0263 (7)	−0.0019 (6)
C2	0.0520 (8)	0.0356 (8)	0.0456 (8)	0.0027 (6)	0.0272 (7)	0.0032 (6)
C3	0.0490 (8)	0.0377 (8)	0.0413 (8)	0.0026 (6)	0.0228 (7)	0.0023 (6)
C4	0.0881 (13)	0.0434 (9)	0.0560 (10)	0.0048 (9)	0.0408 (10)	0.0079 (8)
C5	0.0989 (16)	0.0556 (11)	0.0519 (10)	0.0053 (10)	0.0425 (11)	0.0125 (8)
C6	0.0718 (12)	0.0775 (13)	0.0489 (10)	−0.0054 (10)	0.0360 (9)	−0.0048 (9)
C7	0.0591 (10)	0.0553 (10)	0.0441 (9)	0.0095 (8)	0.0232 (8)	−0.0048 (7)
C8	0.0502 (8)	0.0471 (9)	0.0409 (8)	0.0059 (7)	0.0198 (7)	−0.0012 (6)
C9	0.0474 (9)	0.0705 (12)	0.0529 (10)	0.0028 (8)	0.0229 (8)	0.0019 (8)
C10	0.0480 (9)	0.0863 (14)	0.0494 (10)	−0.0035 (9)	0.0120 (8)	−0.0056 (9)
C11	0.0776 (12)	0.0604 (11)	0.0467 (10)	−0.0109 (9)	0.0242 (9)	−0.0126 (8)
C12	0.0768 (13)	0.0928 (16)	0.0453 (10)	0.0270 (12)	0.0172 (10)	0.0131 (10)
C13	0.0617 (9)	0.0335 (8)	0.0406 (8)	0.0006 (6)	0.0282 (7)	0.0013 (6)
C14	0.0665 (11)	0.0519 (10)	0.0701 (12)	−0.0058 (8)	0.0365 (10)	0.0029 (8)
C15	0.1005 (17)	0.0571 (13)	0.0889 (16)	−0.0289 (12)	0.0497 (14)	−0.0048 (11)
C16	0.141 (2)	0.0356 (9)	0.0696 (13)	−0.0053 (12)	0.0546 (15)	0.0023 (9)
C17	0.1080 (17)	0.0449 (10)	0.0599 (11)	0.0206 (11)	0.0419 (12)	0.0118 (8)
C18	0.0689 (11)	0.0469 (9)	0.0502 (9)	0.0084 (8)	0.0292 (8)	0.0051 (7)
C19	0.0726 (12)	0.0502 (10)	0.0541 (10)	−0.0017 (8)	0.0264 (9)	−0.0092 (8)
C20	0.0829 (15)	0.0723 (15)	0.0653 (13)	−0.0105 (11)	0.0325 (11)	−0.0228 (11)
C21	0.132 (2)	0.0667 (14)	0.0634 (13)	−0.0083 (14)	0.0501 (14)	−0.0103 (10)

### Geometric parameters (Å, º)

**Table:**

S1—C1	1.7418 (17)	C9—H9B	0.9700
S1—C19	1.8161 (19)	C10—C12	1.517 (3)
N1—C1	1.370 (2)	C10—C11	1.519 (3)
N1—C2	1.389 (2)	C10—H10A	0.9800
N1—C13	1.4450 (19)	C11—H11A	0.9700
N2—C1	1.302 (2)	C11—H11B	0.9700
N2—N3	1.395 (2)	C12—H12A	0.9700
N3—C2	1.302 (2)	C12—H12B	0.9700
O1A—C20	1.371 (3)	C13—C14	1.374 (3)
O1A—C21	1.430 (3)	C13—C18	1.379 (2)
O1B—C20	1.119 (9)	C14—C15	1.394 (3)
O1B—C21	1.524 (11)	C14—H14A	0.9300
C2—C3	1.507 (2)	C15—C16	1.371 (4)
C3—C4	1.537 (2)	C15—H15A	0.9300
C3—C8	1.538 (2)	C16—C17	1.359 (4)
C3—C9	1.538 (2)	C16—H16A	0.9300
C4—C5	1.534 (3)	C17—C18	1.384 (3)
C4—H4A	0.9700	C17—H17A	0.9300
C4—H4B	0.9700	C18—H18A	0.9300
C5—C12	1.520 (3)	C19—C20	1.491 (3)
C5—C6	1.530 (3)	C19—H19A	0.9700
C5—H5A	0.9800	C19—H19B	0.9700
C6—C7	1.522 (3)	C20—H20A	0.9700
C6—H6A	0.9700	C20—H20B	0.9700
C6—H6B	0.9700	C20—H20C	0.9601
C7—C11	1.518 (3)	C20—H20D	0.9599
C7—C8	1.536 (2)	C21—H21A	0.9599
C7—H7A	0.9800	C21—H21B	0.9600
C8—H8A	0.9700	C21—H21C	0.9600
C8—H8B	0.9700	C21—H21D	0.9600
C9—C10	1.534 (3)	C21—H21E	0.9598
C9—H9A	0.9700	C21—H21F	0.9600
			
C1—S1—C19	98.08 (9)	C10—C12—H12B	109.9
C1—N1—C2	104.57 (13)	C5—C12—H12B	109.9
C1—N1—C13	125.03 (13)	H12A—C12—H12B	108.3
C2—N1—C13	130.39 (13)	C14—C13—C18	120.99 (16)
C1—N2—N3	106.50 (13)	C14—C13—N1	119.80 (15)
C2—N3—N2	108.70 (13)	C18—C13—N1	119.21 (15)
C20—O1A—C21	111.5 (2)	C13—C14—C15	118.7 (2)
C21—O1A—H20C	105.5	C13—C14—H14A	120.7
C20—O1A—H21E	146.8	C15—C14—H14A	120.7
H20C—O1A—H21E	117.0	C16—C15—C14	120.5 (2)
C20—O1B—C21	121.6 (9)	C16—C15—H15A	119.7
C20—O1B—H21B	152.8	C14—C15—H15A	119.7
N2—C1—N1	111.12 (14)	C17—C16—C15	120.0 (2)
N2—C1—S1	126.93 (13)	C17—C16—H16A	120.0
N1—C1—S1	121.95 (12)	C15—C16—H16A	120.0
N3—C2—N1	109.10 (14)	C16—C17—C18	120.8 (2)
N3—C2—C3	123.72 (14)	C16—C17—H17A	119.6
N1—C2—C3	127.16 (14)	C18—C17—H17A	119.6
C2—C3—C4	108.26 (13)	C13—C18—C17	119.01 (19)
C2—C3—C8	111.61 (13)	C13—C18—H18A	120.5
C4—C3—C8	108.03 (14)	C17—C18—H18A	120.5
C2—C3—C9	111.62 (13)	C20—C19—S1	110.44 (15)
C4—C3—C9	108.59 (15)	C20—C19—H19A	109.6
C8—C3—C9	108.64 (14)	S1—C19—H19A	109.6
C5—C4—C3	110.57 (15)	C20—C19—H19B	109.6
C5—C4—H4A	109.5	S1—C19—H19B	109.6
C3—C4—H4A	109.5	H19A—C19—H19B	108.1
C5—C4—H4B	109.5	O1B—C20—O1A	52.9 (7)
C3—C4—H4B	109.5	O1B—C20—C19	136.8 (6)
H4A—C4—H4B	108.1	O1A—C20—C19	112.00 (19)
C12—C5—C6	110.26 (19)	O1B—C20—H20A	114.0
C12—C5—C4	109.43 (18)	O1A—C20—H20A	109.2
C6—C5—C4	109.17 (17)	C19—C20—H20A	109.2
C12—C5—H5A	109.3	O1B—C20—H20B	57.4
C6—C5—H5A	109.3	O1A—C20—H20B	109.2
C4—C5—H5A	109.3	C19—C20—H20B	109.2
C7—C6—C5	108.95 (15)	H20A—C20—H20B	107.9
C7—C6—H6A	109.9	O1B—C20—H20C	103.5
C5—C6—H6A	109.9	O1A—C20—H20C	64.8
C7—C6—H6B	109.9	C19—C20—H20C	102.9
C5—C6—H6B	109.9	H20A—C20—H20C	51.4
H6A—C6—H6B	108.3	H20B—C20—H20C	146.7
C11—C7—C6	109.37 (16)	O1B—C20—H20D	103.0
C11—C7—C8	109.30 (15)	O1A—C20—H20D	145.4
C6—C7—C8	109.82 (15)	C19—C20—H20D	102.4
C11—C7—H7A	109.4	H20A—C20—H20D	53.6
C6—C7—H7A	109.4	H20B—C20—H20D	60.0
C8—C7—H7A	109.4	H20C—C20—H20D	105.0
C7—C8—C3	110.23 (13)	O1A—C21—H21A	109.3
C7—C8—H8A	109.6	O1B—C21—H21A	141.5
C3—C8—H8A	109.6	O1A—C21—H21B	109.6
C7—C8—H8B	109.6	O1B—C21—H21B	68.7
C3—C8—H8B	109.6	H21A—C21—H21B	109.5
H8A—C8—H8B	108.1	O1A—C21—H21C	109.6
C10—C9—C3	109.92 (15)	O1B—C21—H21C	106.9
C10—C9—H9A	109.7	H21A—C21—H21C	109.5
C3—C9—H9A	109.7	H21B—C21—H21C	109.5
C10—C9—H9B	109.7	O1A—C21—H21D	132.2
C3—C9—H9B	109.7	O1B—C21—H21D	109.1
H9A—C9—H9B	108.2	H21A—C21—H21D	64.1
C12—C10—C11	109.68 (18)	H21B—C21—H21D	46.1
C12—C10—C9	109.98 (19)	H21C—C21—H21D	117.2
C11—C10—C9	109.32 (15)	O1A—C21—H21E	65.1
C12—C10—H10A	109.3	O1B—C21—H21E	109.0
C11—C10—H10A	109.3	H21A—C21—H21E	49.2
C9—C10—H10A	109.3	H21B—C21—H21E	144.7
C7—C11—C10	110.02 (16)	H21C—C21—H21E	104.8
C7—C11—H11A	109.7	H21D—C21—H21E	109.5
C10—C11—H11A	109.7	O1A—C21—H21F	117.1
C7—C11—H11B	109.7	O1B—C21—H21F	110.3
C10—C11—H11B	109.7	H21A—C21—H21F	107.4
H11A—C11—H11B	108.2	H21B—C21—H21F	103.8
C10—C12—C5	109.14 (16)	H21D—C21—H21F	109.5
C10—C12—H12A	109.9	H21E—C21—H21F	109.5
C5—C12—H12A	109.9		
			
C1—N2—N3—C2	−0.2 (2)	C2—C3—C9—C10	−177.38 (15)
N3—N2—C1—N1	0.5 (2)	C4—C3—C9—C10	−58.1 (2)
N3—N2—C1—S1	179.88 (13)	C8—C3—C9—C10	59.14 (19)
C2—N1—C1—N2	−0.63 (18)	C3—C9—C10—C12	60.3 (2)
C13—N1—C1—N2	178.41 (15)	C3—C9—C10—C11	−60.2 (2)
C2—N1—C1—S1	179.98 (12)	C6—C7—C11—C10	60.1 (2)
C13—N1—C1—S1	−1.0 (2)	C8—C7—C11—C10	−60.1 (2)
C19—S1—C1—N2	0.81 (19)	C12—C10—C11—C7	−60.1 (2)
C19—S1—C1—N1	−179.90 (14)	C9—C10—C11—C7	60.6 (2)
N2—N3—C2—N1	−0.18 (19)	C11—C10—C12—C5	59.3 (2)
N2—N3—C2—C3	178.35 (15)	C9—C10—C12—C5	−60.9 (2)
C1—N1—C2—N3	0.48 (17)	C6—C5—C12—C10	−59.7 (2)
C13—N1—C2—N3	−178.49 (16)	C4—C5—C12—C10	60.4 (2)
C1—N1—C2—C3	−177.99 (15)	C1—N1—C13—C14	−95.7 (2)
C13—N1—C2—C3	3.0 (3)	C2—N1—C13—C14	83.1 (2)
N3—C2—C3—C4	−7.1 (2)	C1—N1—C13—C18	83.7 (2)
N1—C2—C3—C4	171.18 (16)	C2—N1—C13—C18	−97.5 (2)
N3—C2—C3—C8	−125.84 (17)	C18—C13—C14—C15	2.3 (3)
N1—C2—C3—C8	52.4 (2)	N1—C13—C14—C15	−178.32 (18)
N3—C2—C3—C9	112.38 (18)	C13—C14—C15—C16	−0.3 (3)
N1—C2—C3—C9	−69.4 (2)	C14—C15—C16—C17	−1.4 (4)
C2—C3—C4—C5	179.69 (16)	C15—C16—C17—C18	1.1 (3)
C8—C3—C4—C5	−59.3 (2)	C14—C13—C18—C17	−2.6 (3)
C9—C3—C4—C5	58.3 (2)	N1—C13—C18—C17	178.02 (15)
C3—C4—C5—C12	−60.0 (2)	C16—C17—C18—C13	0.9 (3)
C3—C4—C5—C6	60.8 (2)	C1—S1—C19—C20	179.61 (15)
C12—C5—C6—C7	59.8 (2)	C21—O1B—C20—O1A	44.6 (9)
C4—C5—C6—C7	−60.4 (2)	C21—O1B—C20—C19	127.7 (7)
C5—C6—C7—C11	−59.5 (2)	C21—O1A—C20—O1B	−43.2 (7)
C5—C6—C7—C8	60.5 (2)	C21—O1A—C20—C19	−176.1 (2)
C11—C7—C8—C3	59.56 (19)	S1—C19—C20—O1B	8.4 (12)
C6—C7—C8—C3	−60.43 (19)	S1—C19—C20—O1A	67.0 (2)
C2—C3—C8—C7	177.63 (14)	C20—O1A—C21—O1B	34.6 (6)
C4—C3—C8—C7	58.74 (19)	C20—O1B—C21—O1A	−49.5 (9)
C9—C3—C8—C7	−58.88 (18)		
